# Basic psychological needs and parental bonding in Italian adults at high risk of hikikomori (extreme social withdrawal): the distinctive association of Competence Frustration with symptom severity

**DOI:** 10.3389/fpsyg.2025.1738750

**Published:** 2026-01-21

**Authors:** Virginia Pupi, Cinzia Bressi, Paolo Brambilla, Maria Gloria Rossetti, Cinzia Perlini, Francesca Girelli, Maria Diletta Buio, Niccolò Zovetti, Isabella Fanizza, Antonio Trabacca, Fabrizia Claudia Guarnieri, Roberto Sassi, Marcella Bellani, Antonella Delle Fave

**Affiliations:** 1 Department of Pathophysiology and Transplantation, University of Milan, Milan, Italy; 2 Department of Neurosciences and Mental Health, Fondazione IRCCS Ca' Granda Ospedale Maggiore Policlinico, Milan, Italy; 3 Section of Psychiatry, Department of Neurosciences, Biomedicine and Movement Sciences, University of Verona, Verona, Italy; 4 Unit of Psychiatry, Azienda Ospedaliera Universitaria Integrata (AOUI) Verona, Verona, Italy; 5 Unit for Severe Disabilities in Developmental Age and Young Adults, Scientific Institute, IRCCS E. Medea, Brindisi, Italy; 6 Scientific Institute IRCCS Eugenio Medea, Scientific Direction, Bosisio Parini, Lecco, Italy; 7 Institute of Neuroscience, National Research Council (CNR), Vedano al Lambro (MB), Italy; 8 Department of Computer Science, University of Milan, Milan, Italy

**Keywords:** basic psychological needs, Competence Frustration, extreme social withdrawal, hikikomori, parental bonding

## Abstract

This brief research report explored the relationships between hikikomori symptom severity (extreme social withdrawal), basic psychological needs of Competence, Autonomy, and Relatedness, and perceived parental bonding in Italian adults experiencing social isolation. Participants (*N* = 33; *M*age = 27.83, *SD* = 7.46; 42.9% women) were individuals recruited online who scored above the high-risk cutoff for hikikomori on the *Hikikomori Questionnaire-25* (HQ-25). They completed the *Basic Psychological Need Satisfaction and Frustration Scale* (BPNSFS) and the *Parental Bonding Instrument* (PBI). Competence Frustration accounted for substantial variability in hikikomori symptom severity in this high-risk sample, explaining approximately 31% of the variance. Regarding perceived parental bonding, maternal Care was positively associated with Autonomy Satisfaction and negatively with Competence Frustration, whereas maternal Control was positively related to frustration of all three needs. Paternal Care was negatively related to Autonomy and Competence Frustration, while paternal Control was positively associated with Relatedness Frustration. Over 30% of participants perceived maternal bonding as Affectionless Control and paternal bonding as either Affectionless Control or Neglectful. No gender differences emerged. Findings suggest that Competence Frustration may represent a key psychological correlate of hikikomori symptom severity in this high-risk group. Moreover, distinct maternal and paternal patterns of perceived Care and Control were associated with need frustration and satisfaction, as well as with the hikikomori dimension of perceived lack of Emotional Support. Study limitations include small sample size, cross-sectional design, reliance on self-report measures, and potential selection bias toward help-seeking individuals. Replication in larger longitudinal samples is warranted to confirm these preliminary results.

## 1 Introduction

Hikikomori (or extreme social withdrawal) refers to a voluntary and prolonged self-isolation at home for at least 6 months (Kato et al., 2019). Primarily involving adolescents and young adults, it can be associated with other comorbidities and has significant implications for individuals' development and psychological well-being (Wang et al., 2013). Originally considered a culture-bound syndrome typical of Japanese society, where it was first described and labeled as *hikikomori* (Saitō, 2012), the phenomenon is now reported across countries and continents (e.g., Malagón-Amor et al., 2015; Hamasaki et al., 2022; Pupi et al., 2025).

Vulnerability to hikikomori seems to be related to specific family and cultural features. Extensive research has shown that parenting styles characterized by overprotection, surveillance, or rejection can exacerbate social withdrawal, disengagement, and communication difficulties (Ike et al., 2020; Li et al., 2020). In Japan, hikikomori has been associated with specific family patterns, such as *amae*, an indulgent maternal style that reinforces dependence (Doi, 1973), and with limited or absent paternal involvement in the child's education (Kato et al., 2019). Although these dynamics reflect Japanese specific practices, comparable parenting styles have been observed across different countries and cultural contexts, where they may similarly hinder adolescents' adaptive development toward adulthood and their fulfillment of personal and social needs.

As specifically concerns need fulfillment, international studies based on Self-Determination Theory (SDT; La Guardia, 2009; Luyckx et al., 2009) have highlighted that satisfaction of the three basic psychological needs (BPNs) for Autonomy, Competence, and Relatedness is fundamental for adaptive identity development during the transition to adulthood and for well-being across the life span. Autonomy refers to the extent to which individuals experience a sense of volition and responsibility for their own behavior. Competence concerns the extent to which individuals feel effective in their social interactions and perceive opportunities to express and develop their abilities. Finally, Relatedness is defined as the extent to which individuals experience a secure sense of belonging and connectedness with others in their social context (Ryan and Deci, 2002). Notably, frustration of these needs does not represent the mere lack of satisfaction, but an experience of pressure, ineffectiveness, or exclusion that undermines adaptation (Bartholomew et al., 2011; Chen et al., 2015). From a developmental perspective, parental behaviors play a key role in fostering or hindering needs fulfillment. Overly protective and directive parents tend to restrict children's autonomy, discourage their independence, and control their activities (Rubin et al., 2009). As a result, children may not develop adaptive coping and problem-solving strategies at both the individual and relational levels. While parental overprotectiveness and control have been associated with children's social withdrawal (e.g., Coplan et al., 2004; Lieb et al., 2000), little is known about how perceived parental care and control relate to basic psychological need satisfaction and frustration in individuals exhibiting hikikomori symptoms. This exploratory study aimed to address this gap by examining the interplay between hikikomori symptom severity, basic psychological need satisfaction and frustration, and perceived parental bonding in an Italian sample of adults at high risk of hikikomori, with a specific focus on factors associated with variability in symptom severity.

## 2 Materials and methods

### 2.1 Participants and procedure

Participants were 33 adults aged 18–47 years (*M* = 27.83, *SD* = 7.46; 42.9% women), recruited through social media announcements and flyers advertising free psychotherapy sessions for mental well-being and social skills. This recruitment strategy reflects the difficulty of accessing individuals at high risk of hikikomori, who constitute a hard-to-reach population, and was therefore expected to capture a help-seeking subgroup. The invitation targeted young adults experiencing social isolation and interested in improving their social life and emotional well-being. Eligibility criteria included scoring above the cutoff value indicating high risk of hikikomori on the *Hikikomori Questionnaire-25* (HQ-25; Teo et al., 2018; Italian version: Amendola et al., 2022). The sessions were offered as part of the *SOLITAIRE* study (ClinicalTrials.gov ID: NCT06138301), a national multicentric research project funded by the European Union—Next Generation EU—PNRR M6C2—Investment 2.1 “Enhancement and strengthening of biomedical research in the NHS” (2022). The study was conducted within a consortium of academic and clinical institutions aiming to investigate different aspects of social isolation in youth, and to test the effectiveness of multimodal interventions, including telepsychotherapy (Rossetti et al., 2024) and cognitive training on participants' mental health. Participation was voluntary and based on written informed consent. Anonymity was ensured through pseudonymization procedures. Ethical approval for the study was granted by the Ethics Committee of the Azienda Ospedaliera Universitaria Integrata of Verona, Verona, Italy.

### 2.2 Measures

The *Hikikomori Questionnaire-25* (HQ-25; Teo et al., 2018; Italian version Amendola et al., 2022) is a 25- item self-report instrument, assessing the severity of hikikomori symptoms over the preceding 6 months. Symptoms are grouped into three dimensions, corresponding to three HQ subscales: Socialization (e.g., “*I stay away from other people*”), Isolation (e.g. “*I spend most of my time at home*”), and Emotional Support (e.g., “*There really is not anyone very significant in my life*”). Items are rated on a 5-point Likert scale (0 = “strongly disagree” to 4 = “strongly agree”), with a HQ total score range of 0–100; higher values indicate more severe symptomatology. A cutoff score of 42 on the HQ-25 identifies individuals at high risk of hikikomori, with a sensitivity of 94% and a specificity of 61% (Teo et al., 2018). In the present study, good internal consistency was detected for the total scale (α = 0.79) and the Socialization subscale (α = 0.85). By contrast, internal consistency was marginal for Isolation and Emotional Support (α = 0.65 and α = 0.64, respectively). Alpha values in the 0.60–0.70 range are generally considered acceptable in early-stage, group-level research (Nunnally and Bernstein, 1994). Accordingly, the reliability values observed in the present sample were deemed acceptable, although findings involving the Isolation and Emotional Support subscales were interpreted with caution due to potential measurement error.

The *Basic Psychological Need Satisfaction and Frustration Scale* (BPNSFS; Chen et al., 2015; Italian version Costa et al., 2018) includes 24 items assessing satisfaction and frustration of the three psychological needs in daily life: Autonomy Satisfaction (4 items; e.g., “*I feel a sense of choice and freedom in the things I undertake*”), Competence Satisfaction (4 items; e.g., “*I feel confident that I can do things well*”), Relatedness Satisfaction (4 items; e.g., “*I feel that the people I care about also care about me*”), Autonomy Frustration (4 items; e.g., “*I feel forced to do many things I wouldn't choose to do*”), Competence Frustration (4 items; e.g., “*I have serious doubts about whether I can do things well*”), and Relatedness Frustration (4 items; e.g., “*I feel that people who are important to me are cold and distant towards me*”). Responses are rated on a 5-point Likert scale (ranging from 1 = “completely disagree” to 5 = “completely agree”). Internal consistency was α = 0.76 for Autonomy Satisfaction, α = 0.93 for Competence Satisfaction, α = 0.88 for Relatedness Satisfaction, α = 0.75 for Autonomy Frustration, α = 0.90 for Competence Frustration, α = 0.83 for Relatedness Frustration.

The *Parental Bonding Instrument* (PBI; Parker et al., 1979; Italian version Favaretto et al., 2001) is a 25- item self-report questionnaire assessing participants perceived parental behaviors during their first 16 years of life. It comprises two dimensions: Care (12 items; range 0–36), with high scores indicating warmth and acceptance; and Control/Overprotection (13 items; range 0–39), with high scores indicating parental control and restriction of autonomy. Participants rate each parent separately on a 4-point Likert scale (0 = “very unlikely” to 3 = “very likely”). This study follows Favaretto et al. (2001) in both the use of “Care” and “Control” dimensions, conceptually corresponding to Care and Overprotection in the original framework (Parker et al., 1979), and the cutoff values defined for these two dimensions. Specifically, scores of maternal Care ≥ 27 and paternal Care ≥ 24 indicate high levels of care, whereas maternal Control ≥ 13.5 and paternal Control ≥ 12.5 indicate high levels of control. By combining Care and Control scores based on these cutoff values, four parenting styles can be identified: Affectionate Constraint (high care, high control), Affectionless Control (low care, high control), Optimal parenting (high care, low control), and Neglectful Parenting (low care, low control). Consistent with the PBI's two-factor structure, we treated Care and Control as two subscales with separate total scores. Internal consistency was α = 0.82 for maternal Care, α = 0.87 for maternal Control, α = 0.95 for paternal Care, and α = 0.87 for paternal Control.

### 2.3 Data analysis

Descriptive statistics were computed for sociodemographic, clinical, and study variables. As one participant did not provide valid responses to the PBI, analyses for this instrument were conducted on 32 participants. Independent-samples *t-*tests were performed to examine gender differences in hikikomori symptom severity (HQ-25), need satisfaction and frustration (BPNSFS), and perceived parental care and control (PBI). Additional *t*-tests were conducted to compare hikikomori symptom severity between participants with high vs. low levels of perceived parental Care and Control, based on PBI cutoff scores. Bivariate Pearson correlations were calculated to explore associations between parental bonding dimensions, basic psychological need satisfaction and frustration, and hikikomori symptoms (total and subscale scores). Given the small sample size, *post-hoc* power levels for the main correlation analyses were computed and are reported in the Supplementary Tables S1, S2.

Finally, a standard multiple regression analysis was conducted to examine factors associated with variability in overall hikikomori symptom severity within this high-risk group. Considering the limited sample size, the regression model was specified using a deliberately conservative approach to reduce the risk of overfitting and unstable estimates. Predictor selection followed a hierarchical procedure.

First, candidate predictors were identified based on significant bivariate correlations with hikikomori symptom severity (Table 4). Second, among these empirically associated variables, theoretical considerations grounded in Self-Determination Theory were applied, prioritizing indicators of basic psychological need frustration as proximal vulnerability processes and need satisfaction as potential protective resources supporting adaptive functioning (Deci and Ryan, 2000; Vansteenkiste and Ryan, 2013). Third, highly collinear constructs were excluded to preserve parsimony and interpretability. Accordingly, Competence Frustration and Autonomy Satisfaction were selected as predictors, whereas Competence Satisfaction was excluded due to conceptual and empirical overlap with Competence Frustration. Due to the small sample size, *post-hoc* power for the regression model was also computed.

Statistical analyses were performed using SPSS version 25, with a significance threshold of *p* < 0.05.

## 3 Results

### 3.1 Hikikomori symptom severity

In line with the inclusion criteria, all participants scored above the HQ-25 clinical cut-off (>42; Teo et al., 2018), indicating a high risk of hikikomori, with no significant gender differences (men *M* = 66.47, *SD* = 12.55; women *M* = 61.93, *SD* = 9.34), *t*_(31)_ = 1.14, *p* = 0.26, Cohen's *d* = 0.37. As for clinical background, 17 participants (51.5%) were users of psychiatric services at the time of the study, 15 (45.5%) were not, and one did not respond (3%). Previous psychotherapy had been undertaken by 18 participants (54.5%); 11 (33.3%) reported no prior treatment experience, and 4 (12.1%) did not provide this information. The most frequently reported psychiatric comorbidities were anxiety combined with mood disorders (*n* = 10, 30.3%) and mood disorders alone (*n* = 7, 21.2%). Few participants mentioned personality disorders (*n* = 1, 3.0%), psychosis (*n* = 1, 3.0%), and various combinations of anxiety, mood, PTSD, ADHD, and eating disorders (*n* = 5, 15.1%). Finally, 3 participants (9.1%) reported no psychiatric comorbidity, and 6 (18.2%) did not provide this information.

### 3.2 Basic psychological needs and perceived parental bonding

Table 1 shows descriptive statistics for participants' basic need satisfaction and frustration and perceived maternal and paternal Care and Control by gender. Frequencies of perceived parental bonding are presented in Table 2. Results on parental bonding refer to 32 participants, as one did not provide valid responses to the PBI.

**Table 1:** Means (*M*) and standard deviations (*SD*) of the Basic Psychological Need Satisfaction and Frustration Scale (BPNSFS) dimensions by gender (*N* = 33) and the Parental Bonding Instrument (PBI) dimensions by gender (*N* = 32).

**Variable**	**Men (*n* = 19)**	**Women (*n* = 14)**
**BPNSFS**		
Autonomy satisfaction	11.26 (3.36)	10.93 (2.97)
Competence satisfaction	11.79 (3.84)	11.36 (5.18)
Relatedness satisfaction	13.37 (3.82)	12.93 (3.32)
Autonomy frustration	11 (4.20)	12.14 (2.74)
Competence frustration	12.21 (4.67)	13.14 (4.91)
Relatedness frustration	12.11 (4.55)	11.86 (3.88)
**PBI**		
Maternal care	23.44 (6.91)	23.57 (4.59)
Maternal control	15.11 (8.53)	15.29 (7.55)
Paternal care	14.17 (8.49)	20.57 (9.64)
Paternal control	13.67 (9.88)	10.79 (3.66)

BPNSFS, Basic Psychological Need Satisfaction and Frustration Scale; PBI, Parental Bonding Instrument. For the PBI, higher Care scores indicate greater parental warmth, whereas higher Control scores indicate greater parental overprotection. Italian cutoffs were used to identify high levels of Care (maternal ≥ 27; paternal ≥ 24) and high levels of Control (maternal ≥ 13.5; paternal ≥ 12.5), separately for mothers and fathers (Favaretto et al., 2001).

**Table 2:** Frequencies and percentages of perceived parenting styles based on the *Parental Bonding Instrument* (*N* = 32).

**Parenting style**	**Maternal parenting style**	**Paternal parenting style**
Affectionate constraint	3 (9.4%)	1 (3.1%)
Affectionless control	13 (40.6 %)	11 (34.4%)
Optimal parenting	9 (28.1 %)	8 (25.0%)
Neglectful parenting	7 (21.9%)	12 (37.5%)

Parenting styles were derived by combining PBI Care and Control dimensions based on Italian cutoffs (Favaretto et al., 2001): Affectionate Constraint = high care, high control; Affectionless Control = low care, high control; Optimal parenting = high care, low control; Neglectful Parenting = low care, low control.

As regards need satisfaction and frustration, independent samples *t*-tests revealed no significant gender differences across BPNSFS dimensions, as all *p*-values were higher than 0.35. Although unequal variances were detected for Autonomy Frustration (*p* = 0.006), Welch's correction confirmed the non-significant result (*p* = 0.35).

With reference to parental bonding, both maternal and paternal Care scores were below the respective Italian cutoffs for high care, whereas maternal and paternal Control scores were above the thresholds for high control, except for paternal Control in women participants. Independent-samples *t*-tests did not highlight significant gender differences in maternal Care [*t*_(30)_ = 0.06, *p* = 0.95], maternal Control [*t*_(30)_ = 0.06, *p* = 0.95], and paternal Control [*t*_(30)_ = −1.03, *p* = 0.31]. A trend-level effect [*t*_(30)_ = 2.00, *p* = 0.055] instead emerged for paternal Care, with higher values reported by women (*M* = 20.57, *SD* = 9.64) compared to men (*M* = 14.17, *SD* = 8.49).

Additional independent-samples *t*-tests were conducted to assess differences in hikikomori symptom severity as a function of high vs. low levels of maternal and paternal Care and Control. No significant differences were found across comparisons (all *ps* > 0.09). More specifically, hikikomori symptom severity did not significantly differ between participants reporting low maternal Care (*M* = 65.75, *SD* = 11.37, *n* = 20) and high maternal Care (*M* = 63.33, *SD* = 11.84, *n* = 12), *t*_(30)_ = 0.57, *p* = 0.571, 95% CI (−6.19, 11.02), *d* = 0.21. Although participants with low paternal Care showed higher HQ-25 scores (*M* = 66.52, *SD* = 11.69, *n* = 23) than those with high paternal Care (*M* = 59.00, *SD* = 9.62, *n* = 9), the difference was not statistically significant, *t*_(30)_ = 1.71, *p* = 0.097, 95% CI (−1.45, 16.50), *d* = 0.67. Similarly, no significant differences were observed for maternal Control (low: *M* = 63.38, *SD* = 9.60, *n* = 16; high: *M* = 66.31, *SD* = 13.14, *n* = 16) *t*_(30)_ = −0.72, *p* = 0.476, 95% CI (−11.25, 5.37), *d* = −0.25, or paternal Control (low: *M* = 65.42, *SD* = 10.66, *n* = 19; high: *M* = 62.92, *SD* = 12.97, *n* = 13), *t*_(30)_ = 0.60, *p* = 0.556, 95% CI (−6.06, 11.06), *d* = 0.21.

### 3.3 Correlations between basic psychological needs, perceived parental bonding, and hikikomori symptom severity

Pearson correlations were performed to examine the associations between individuals' BPNs satisfaction and frustration and perceived parental bonding dimensions (Table 3). Correlations were also calculated between hikikomori symptom severity (total and subscale scores), basic psychological need satisfaction and frustration, and parental bonding dimensions (Table 4).

**Table 3:** Pearson correlations between basic psychological need satisfaction and frustration (BPNSFS) and parental bonding dimensions (PBI).

**Variable**	**Maternal care**	**Maternal control**	**Paternal care**	**Paternal control**
Autonomy satisfaction	0.42^*^	−0.15	0.23	−0.23
Competence satisfaction	0.07	−0.04	0.15	−0.11
Relatedness satisfaction	0.24	−0.32	0.25	−0.22
Autonomy frustration	−0.31	0.37^*^	−0.38^*^	0.33
Competence frustration	−0.41^*^	0.41^*^	−0.36^*^	0.21
Relatedness frustration	−0.22	0.47^**^	−0.33	0.38^*^

Values represent Pearson's *r*. BPNSFS, Basic Psychological Need Satisfaction and Frustration Scale; PBI, Parental Bonding Instrument. ^*^p < 0.05; ^**^p < 0.01; *N* = 33 for correlations excluding PBI; *N* = 32 for correlations including PBI.

**Table 4:** Pearson correlations of hikikomori symptom severity (HQ-25; total and subscale scores) with basic psychological need satisfaction and frustration (BPNSF) and parental bonding dimensions (PBI).

**Variable**	**Hikikomori symptom severity (HQ-25 total score)**	**Socialization**	**Isolation**	**Emotional support**
Autonomy satisfaction	−0.43^*^	−0.58^**^	−0.23	0.16
Competence satisfaction	−0.36^*^	−0.32	−0.28	−0.09
Relatedness satisfaction	−0.33	−0.10	−0.23	−0.47^**^
Autonomy frustration	0.20	0.13	0.07	0.23
Competence frustration	0.54^**^	0.42^*^	0.34	0.32
Relatedness frustration	0.27	−0.12	0.30	0.62^**^
Maternal care	−0.30	−0.19	−0.22	−0.25
Maternal control	0.26	0.11	0.04	0.45^**^
Paternal care	−0.29	−0.09	−0.17	−0.42^*^
Paternal control	0.23	0.06	0.01	0.49^**^

Values represent Pearson's *r*. BPNSFS, Basic Psychological Need Satisfaction and Frustration Scale; HQ-25, Hikikomori Questionnaire−25; PBI, Parental Bonding Instrument. ^*^p < 0.05; ^**^p < 0.01; *N* = 33 for correlations excluding PBI; *N* = 32 for correlations including PBI.

Results showed that maternal Care was positively correlated with Autonomy Satisfaction and negatively correlated with Competence Frustration. Maternal Control was positively associated with frustration of all BPNs (Autonomy, Competence, and Relatedness). Paternal Care was negatively correlated with Autonomy and Competence Frustration, while paternal Control showed a positive association with Relatedness Frustration.

As shown in Table 4, hikikomori symptom severity (HQ-25 total score) was negatively correlated with Autonomy and Competence satisfaction, and positively with Competence Frustration. As for hikikomori symptom subscales, lack of Socialization was negatively correlated with Autonomy Satisfaction and positively with Competence Frustration, while lack of Emotional Support subscale was negatively correlated with Relatedness Satisfaction and positively with Relatedness Frustration.

Significant correlations between hikikomori symptom severity and perceived parental bonding dimensions emerged only for lack of Emotional Support, which was positively associated with both maternal and paternal Control and negatively with paternal Care.

### 3.4 Predictors of hikikomori symptom severity

Based on the analytic strategy described in the Data Analysis section, a multiple regression model was specified to examine variability in overall hikikomori symptom severity (HQ-25 total score). Among basic psychological need dimensions, symptom severity was significantly correlated with Competence Frustration, Autonomy Satisfaction, and Competence Satisfaction (Table 4).

Competence Frustration was included as an indicator of vulnerability, given its association with hikikomori symptom severity and previous evidence linking competence frustration to maladaptive and withdrawal-related outcomes, such as depressive symptoms, loneliness, and disengagement (Liu et al., 2025; Saricali et al., 2022). Autonomy Satisfaction was retained to capture potential protective variance, in line with Self-Determination Theory formulations linking autonomy satisfaction to volitional functioning and psychological well-being (Deci and Ryan, 2000; Vansteenkiste and Ryan, 2013). Although Competence Satisfaction was also significantly correlated with hikikomori symptom severity, it was not included in the model due to its strong empirical and conceptual overlap with Competence Frustration, as reflected in their high intercorrelation (*r* = −0.73, *p* < 0.001) and in the moderate association between Competence Satisfaction and Autonomy Satisfaction (*r* = 0.57, *p* < 0.001; see Table 4). Including multiple competence-related indicators would have compromised model stability and interpretability in this small sample.

Although Competence Frustration and Autonomy Satisfaction were moderately intercorrelated (r = −0.58, *p* < 0.001), both were retained as conceptually distinct indicators of basic psychological need functioning. The overall model was statistically significant, *F*_(2, 30)_ = 6.69, *p* = 0.004, explaining approximately 31% of the variance in hikikomori symptom severity within this high-risk group (R^2^ = 0.31, adjusted R^2^ = 0.26), with adequate observed power (0.91). Competence Frustration emerged as a significant positive predictor of hikikomori symptom severity (B = 1.05, SE = 0.45, β = 0.44, *p* = 0.025), whereas Autonomy Satisfaction was not significantly associated with the outcome (B = −0.63, SE = 0.67, β = −0.17, *p* = 0.36).

## 4 Discussion

The present study explored the associations among hikikomori symptom severity, basic psychological need satisfaction and frustration, and perceived parental bonding in a small sample of Italian adults classified as being at high risk for hikikomori. Given the hard-to-reach nature of this population, online recruitment resulted in a specific subgroup characterized by elevated psychological distress and help-seeking motivation. In line with the study aims, the sample was not intended to be representative of hikikomori risk in the general population, but to capture variability in symptom severity among adults screened as being at high risk of hikikomori. Accordingly, only individuals meeting the HQ-25 high-risk cutoff were included. Consistent with available literature, most participants reported psychiatric comorbidities, particularly anxiety and mood disorders, as well as the use of mental health services. Despite its exploratory nature, the study provided meaningful findings.

First, no gender differences emerged in hikikomori symptom severity and in BPNs satisfaction and frustration. On average, both maternal and paternal Care scores were below the Italian cutoffs for high care, whereas maternal and paternal Control values were above the thresholds for high control, except for paternal Control among women. Over one-third of the participants perceived their maternal style as Affectionless Control and their paternal style as either Affectionless Control or Neglectful Parenting.

Second, correlational analyses revealed a positive association between maternal Care and Satisfaction of Autonomy, whereas higher paternal Care co-occurs with lower frustration in the same domain. In addition, higher perceived parental Care was associated with lower Frustration of Competence. Conversely, the perception of higher maternal Control was associated with greater frustration of all basic psychological needs, whereas higher paternal Control was specifically related to Frustration of the need for Relatedness.

As for hikikomori symptoms, their severity was inversely associated with Satisfaction of the needs for Autonomy and Competence and positively associated with Competence Frustration. Specifically, Socialization difficulties were associated with lower Autonomy Satisfaction and higher Competence Frustration. Lack of Emotional Support was closely linked to Relatedness Frustration and lower Satisfaction in this domain. Differently from the other hikikomori components, Isolation was unrelated to need frustration and satisfaction.

Moreover, lack of Emotional Support emerged as the only hikikomori symptom dimension associated with perceived parental bonding: higher scores on this subscale were positively associated with both maternal and paternal Control, and negatively with paternal Care.

Finally, regression analysis showed that Competence Frustration was significantly associated with hikikomori symptom severity, accounting for nearly one-third of the variance. Although observed *post-hoc* power for the model was adequate, this result should be interpreted as preliminary and sample-specific, reflecting an association within a small, highly selected high-risk group rather than evidence of a generalizable or causal predictor.

This result highlights that feeling ineffective and doubtful about one's abilities may constitute a psychological mechanism associated with greater hikikomori symptom severity in this high-risk group of participants. It may reflect a potential vulnerability factor deserving longitudinal examination. In addition, the negative correlation of Autonomy Satisfaction with hikikomori symptom severity suggests the potential link of higher autonomy to less severe withdrawal symptoms, a tendency that warrants further investigation.

From a Self-Determination Theory perspective, the prominent role of competence frustration suggests that interventions aimed at enhancing perceived competence and self-efficacy (e.g., graded skill-building or social competence training) may be particularly relevant for individuals at high risk of hikikomori. Clinically, assessing basic psychological need frustration together with family relational patterns may support more tailored intervention strategies. Finally, examining hikikomori in the Italian context contributes to the growing evidence that this phenomenon extends beyond its original Japanese setting, thus requiring further research in Western sociocultural contexts.

### 4.1 Limitations and future directions

The major limitations of this study are the small sample size and the cross-sectional design, which reduce the statistical power and the generalizability of the findings. Recruitment through advertisements offering free psychotherapy likely favored the inclusion of a help-seeking subgroup characterized by higher psychological distress and treatment motivation, thereby introducing a selection bias. In addition, the sample was restricted to individuals scoring above the HQ-25 high-risk cutoff; accordingly, the findings pertain to variability in symptom severity within a selected high-risk subgroup rather than to risk for hikikomori in the general population.

Although psychiatric comorbidity was documented, the limited sample size did not allow for investigating the relationships of different psychopathological profiles with perceived parental bonding and basic psychological need satisfaction or frustration. Future studies with larger samples should explore these associations while taking into account participants' clinical profiles. Moreover, the exclusive reliance on self-report measures may have introduced biases related to subjective perception, including social desirability and recall bias; this limitation is particularly relevant for parental bonding, which relies on retrospective evaluation of early family experiences.

The small sample size substantially constrained statistical power (see Supplementary materials), increasing the risk of Type II errors and limiting the precision and stability of effect size estimates. In addition, effect sizes may be upwardly biased in small samples, particularly for statistically significant findings. Accordingly, non-significant results should not be interpreted as evidence of absence of association, and statistically significant findings, including those from the regression analysis, should be considered preliminary and aimed at examining associations within this high-risk sample rather than establishing robust or generalizable predictors.

Although the present findings should be interpreted with considerable caution, it is important to consider that hikikomori individuals represent a hard-to-reach population, and in Western societies empirical research has so far been largely limited to case reports or very small clinical samples (Pupi et al., 2025). Hence, despite its limitations, the present study provides novel information about the variability in symptom severity among adults at high risk of hikikomori outside traditional clinical settings. Future research with larger and longitudinal samples is needed to clarify how distinct maternal and paternal bonding patterns influence the satisfaction or frustration of basic psychological needs—particularly Competence—and, in turn, contribute to mechanisms of risk or protection related to hikikomori symptom severity. Subsequent studies should adopt targeted recruitment strategies to reach socially withdrawn individuals who are not help-seeking, extending investigation beyond narrowly defined high-risk subgroups to include individuals meeting criteria for hikikomori as well as those at earlier stages of risk. Further research should also examine how perceived parental bonding relates to broader parenting styles and family sociocultural values (e.g., norms surrounding autonomy and social assertiveness) to identify context-specific factors relevant to the Italian sociocultural setting.

### 4.2 Conclusions

Overall, these findings suggest that Competence Frustration may play a central role in greater hikikomori symptom severity within a high-risk group, while distinct maternal and paternal patterns of Care and Control appear differentially associated with the satisfaction and frustration of individuals' basic psychological needs. Addressing competence-related difficulties together with family relational dynamics could inform the development of family-based interventions, aimed at limiting the progression and severity of hikikomori symptoms during adolescence and young adulthood.

## Data Availability

The raw data supporting the conclusions of this article will be made available by the authors, without undue reservation.
