# Supplementary material for: Basic psychological needs and parental bonding in Italian adults at high risk of hikikomori (extreme social withdrawal): the distinctive association of Competence Frustration with symptom severity
Source: Front Psychol. 2026 Jan 21;16:1738750. doi: 10.3389/fpsyg.2025.1738750 (PMC12867882; doi:10.3389/fpsyg.2025.1738750)
Supplement: Supplementary file 1 [file Table_1.docx]

**Supplementary materials**

| **Table S1.** Post-hoc power for Pearson correlations between basic psychological need satisfaction and frustration (BPNSFS) and parental bonding dimensions (PBI)*.* | | | | |
| --- | --- | --- | --- | --- |
|  | Maternal Care | Maternal Control | Paternal Care | Paternal Control |
| Autonomy Satisfaction | .69 | .13 | .25 | .25 |
| Competence Satisfaction | .07 | .06 | .13 | .09 |
| Relatedness Satisfaction | .27 | .44 | .25 | .23 |
| Autonomy  Frustration | .42 | .56 | .28 | .46 |
| Competence Frustration | .66 | .66 | .54 | .21 |
| Relatedness Frustration | .23 | .80 | .46 | .59 |
| *Note.* Values represent observed post-hoc power (1 − β) for two-tailed Pearson correlations computed in G*Power (version 3.1.9.4) using α = .05 and the observed correlation coefficients (\|r\|) reported in Table 3 of the main manuscript. BPNSFS = *Basic Psychological Need Satisfaction and Frustration Scale;* PBI = *Parental Bonding Instrument.* | | | | |

| **Table S2.** Post-hoc power for Pearson correlations between basic psychological need satisfaction and frustration (BPNSFS) and parental bonding dimensions (PBI)*.* | | | | |
| --- | --- | --- | --- | --- |
|  | Hikikomori symptom severity (HQ-25 total score) | Socialization | Isolation | Emotional Support |
| Autonomy Satisfaction | .73 | .96 | .25 | .14 |
| Competence Satisfaction | .55 | .45 | .36 | .08 |
| Relatedness Satisfaction | .48 | .09 | .25 | .81 |
| Autonomy Frustration | .20 | .11 | .07 | .25 |
| Competence Frustration | .92 | .70 | .50 | .29 |
| Relatedness Frustration | .33 | .10 | .40 | .77 |
| Maternal  Care | .39 | .18 | -.23 | .28 |
| Maternal  Control | .30 | .09 | .06 | .76 |
| Paternal  Care | .37 | .08 | .15 | .69 |
| Paternal  Control | .25 | .06 | .05 | .84 |
| *Note.* Values represent observed post-hoc power (1 − β) for two-tailed Pearson correlations computed in GPower (version 3.1.9.4) using α = .05 and the observed correlation coefficients (\|r\|) reported in Table 4 of the main manuscript. BPNSFS *= Basic Psychological Need Satisfaction and Frustration Scale;* HQ-25 *= Hikikomori Questionnaire–25;* PBI *= Parental Bonding Instrument.* | | | | |

Post-hoc power estimates are provided for descriptive purposes only and should be interpreted with caution, as post-hoc power is mathematically determined by the observed effect size and does not provide information beyond the p value. Given the small sample size (N = 33; N = 32 for correlations involving the PBI), power is expected to be low for small-to-moderate effects; therefore, non-significant correlations may reflect limited sensitivity (Type II error risk), and statistically significant effects may be upwardly biased.
